# Hormones and B-cell development in health and autoimmunity

**DOI:** 10.3389/fimmu.2024.1385501

**Published:** 2024-04-12

**Authors:** Paola Santana-Sánchez, Ricardo Vaquero-García, María Victoria Legorreta-Haquet, Luis Chávez-Sánchez, Adriana Karina Chávez-Rueda

**Affiliations:** Unidad de Investigación Médica en Inmunología, Unidad Médica de Alta Especialidad (UMAE) Hospital de Pediatría, Centro Médico Nacional Siglo XXI, Instituto Mexicano del Seguro Social, Ciudad de México (CDMX), Mexico

**Keywords:** B cell, lymphopoiesis, immune response, hormones, autoimmunity

## Abstract

The development of B cells into antibody-secreting plasma cells is central to the adaptive immune system as they induce protective and specific antibody responses against invading pathogens. Various studies have shown that, during this process, hormones can play important roles in the lymphopoiesis, activation, proliferation, and differentiation of B cells, and depending on the signal given by the receptor of each hormone, they can have a positive or negative effect. In autoimmune diseases, hormonal deregulation has been reported to be related to the survival, activation and/or differentiation of autoreactive clones of B cells, thus promoting the development of autoimmunity. Clinical manifestations of autoimmune diseases have been associated with estrogens, prolactin (PRL), and growth hormone (GH) levels. However, androgens, such as testosterone and progesterone (P4), could have a protective effect. The objective of this review is to highlight the links between different hormones and the immune response mediated by B cells in the etiopathogenesis of systemic lupus erythematosus (SLE), rheumatoid arthritis (RA), and multiple sclerosis (MS). The data collected provide insights into the role of hormones in the cellular, molecular and/or epigenetic mechanisms that modulate the B-cell response in health and disease.

## Introduction

B cells are part of the immune response and play an important defensive role against pathogens through the production of antibodies and the presentation of antigens to T cells. However, B cells are also involved in the development of autoimmune diseases, in which the response is directed against autoantigens. Generally, the development of B cells begins in the bone marrow (BM) and continues in the spleen, after which they migrate to other peripheral lymphoid organs where they are activated and differentiated into memory and plasma B cells. In this process, there is bidirectional communication between the immune and endocrine systems. This relationship has been studied since the 1940s, when Ungar G. demonstrated that hormone-like components are produced in the spleen and secreted into the circulation (1). In rats with splenectomy, a decrease in the concentration of 20α-dihydroprogesterone was found, delaying the onset of ovulation (2), while a reduction in the number of pregnancies was observed in splenectomized mice (3). In people who underwent splenectomy, a reduction in sexual activity, such as decreased libido, erectile dysfunction, and sexual dissatisfaction, was observed (4), demonstrating that splenectomy can interfere with the reproductive system possibly by interfering with hormonal regulation. Furthermore, the functions of the spleen are also regulated by the hypothalamic-pituitary-ovarian axis, and there may be feedback between endocrine molecules and the migration of leukocytes from the spleen to the ovaries (5). In addition to regulating the reproductive system, sex hormones regulate the development and function of immune response cells. It has been shown that females have the ability to produce more antibodies (6), which increases their resistance to infections (7) and decreases their susceptibility to viral infections (8). However, this increase in the immune response can be harmful when one has a predisposition to develop autoimmunity. In both human and mouse models, it has been shown that some hormones promote the development of autoimmunity, while others inhibit it. To date, there is relevant information on the role that hormones play in the development and activation of T cells during the autoimmunity process (9, 10); however, information on the role that hormones play in the development and activation of B cells is scarce. Therefore, in this review, we describe the role of prolactin (PRL), estrogens, growth hormone (GH), testosterone and progesterone (P4) in the development and activation of B cells as well as their role in pathological processes such as autoimmune diseases. This collection of articles demonstrates the importance of endocrine regulation and the B-cell immune response.

## Development of B cells

The development of B cells (B-2) begins in the BM with hematopoietic precursor cells (HSCs) (11), which differentiate into early lymphoid progenitors (ELPs), and subsequently, common lymphoid progenitors (CLPs). For CLPs to commit to a B-cell lineage, different transcription factors, such as E2A (12), EBF (13), and PAX5 (14), are necessary. The transcription factors IKAROS (15)and PU.1 (16), the chemokine axis CXCL12-CXCR4 (17) and the cytokine BAFF (a key cytokine for the development of B cells) (18) are important for the differentiation of B cells in the BM (19). The different stages of cell maturation can be differentiated by the recombination of genes encoding immunoglobulins (Igs), heavy chain (IgH) and light chain (IgL). Both chains are made up of variable and constant regions; the IgH variable region is formed by the VDJ gene segments, while the IgL is generated from the VJ segments (20). This process is known as V(D)J recombination and is mediated by RAG1/RAG2 (21).

The first stage is the formation of pre-pro-B cells, which are characterized by the B220^+^CD43^+^CD19^-^ phenotype and the expression of the Flt3, Il7, and CD79a lineage genes, although these cells still express myeloid lineage-associated transcription factors such as *Runx2*, *Irf8*, and *Tcf4*. The next stage involves the differentiation of pre-pro-B cells into pro-B cells, which exhibit the B220^+^CD93^+^IgM^−^CD43^+^CD25^-^CD23^-^ phenotype; in this stage, the D-J segments of the IgH chain are rearranged, and the functional V-DJ segment subsequently undergoes a rearrangement. This process results in the synthesis of the IgH chain and its expression on the surface, which is associated with the surrogate light chain (λ5 and Vpre-B), which is known as pre-BCR. The formation of the pre-BCR marks the transition to the pre-B stage. This receptor has two functions: the first is to ensure that two IgH chains with different specificities are not expressed in the same cell; this process is called allelic exclusion, and the second function is to initiate the rearrangement of the VJ genes of the IgL chain. Pre-B cells have a B220^+^CD93^+^IgM^−^CD43^-^CD25^+^CD23^-^ phenotype. After the successful rearrangement and expression of the IgL chain, it associates with the previously synthesized IgH chain to form the BCR (IgM), which is transported to the plasma membrane to form the IgM-Igα (CD79a) Igβ (CD79b) complex and marks the transition to the immature B-cell stage. Immature B cells are characterized by the B220^+^CD93^+^IgM^+^CD43^-^CD25^-^CD23^-^ phenotype. At this stage of immature B-cell development, cell-surface antibodies can bind antigens. In the bone marrow microenvironment in which immature B cells emerge, antigens that engage the BCR are almost always self-antigens, which makes regulation at this stage essential. Ligation of the BCR by self-antigens promotes signaling that triggers regulatory processes to reduce self-reactivity. These processes are collectively known as central tolerance (22–25).

The exit of immature B cells from the BM to the periphery is regulated by the receptor for sphingosine-1-phosphate (S1P). These cells that migrate from the BM are called transitional (T) B cells, which have a short half-life and express CD93. These cells arrive in the spleen as T1 B cells (IgM^high^IgD^low^CD24^+^CD93^+^CD21^-^CD23^-^BAFF-R^+/-^) and differentiate into T2 cells (IgMhighIgDintCD24+CD93+CD21+CD23+BAFF-R^+^), where the interaction of BAFF with its receptor (BAFF-R) sends survival signals, such as the activation of antiapoptotic and proliferation factors, so that they progress from the T1 to the T2 stage and complete their maturation process (26–28). Two types of mature B cells can be found: those found in the marginal zone of the spleen, called marginal zone B cells (MZ-B; CD19^+^CD21^+^IgM^high^IgD^low^CD1^+^), and those located in the follicles, known as B follicular cells (FO-B; CD19^+^CD21^+^IgM^low^IgD^high^CD23^+^). The survival of these cells depends on BAFF (an important cytokine for the development of B cells) and their receptor interaction (29–31).

**Figure 1:**
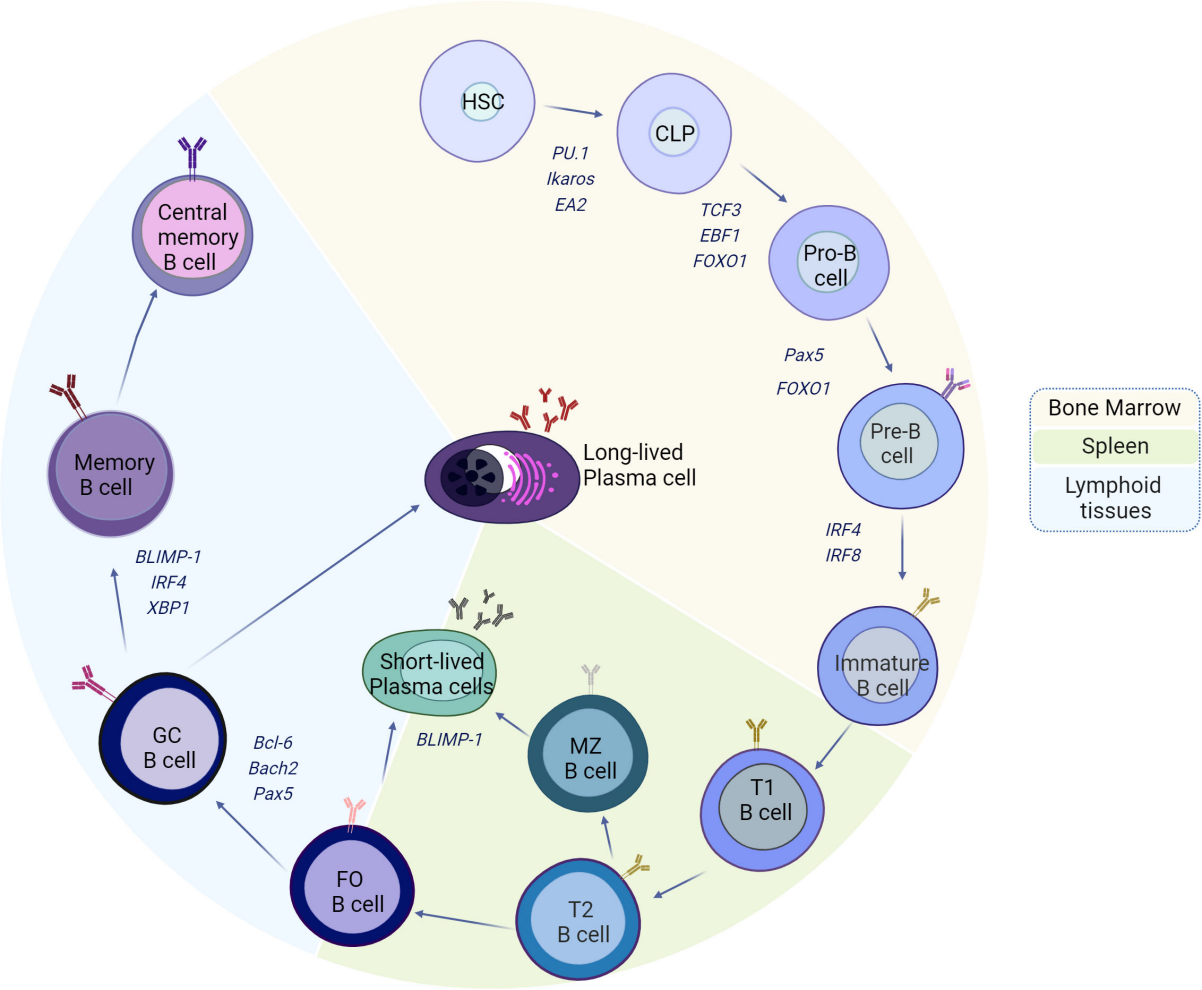
Model of the development and differentiation of B-2 cells and the transcription factors that regulate them. The B-cell ontogeny generally begins in the bone marrow (BM) from hematopoietic stem cells (HSCs), which, through the expression of transcription factors such as PU.1, Ikaros, and EA2, give rise to the formation of common progenitor lymphoid cells. The constant maintenance of EA2, PU.1, and Ikaros, plus the induction of TCF3, EBF1, and FOXO1, promotes commitment to the B-cell lineage, which begins with the mechanisms of immunoglobulin gene recombination and the formation of pre-BCRs (IgH and λ5/VpreB) in pro-B cells. Subsequently, with the VJ rearrangements of the light chains and the expression of Pax5 and FOXO1, pre-B cells emerge from the BM with the stage of immature cells, characterized by the expression of IRF4 and IRF8. The immature cells migrate to the spleen, and during this stage, they go through transitional cell stages 1 (T1) and 2 (T2) to finally reach the marginal zone (MZ) or the B follicles as mature cells. The activation of MZ-B or follicular B cells in the absence of cooperation with follicular T cells (TFH) leads to the formation of short-lived plasma cells, the process of which is highly regulated by BLIMP-1. When follicular B cells are activated in the presence of TFH and IL-21, the expression of Bcl-6 occurs, which, together with Bach2 and Pax5, induces the differentiation of GC-B cells. The maintenance of the activation of GC-B cells, coupled with the maturation of the affinity and increase of BLIMP-1, IRF4, and XBP1, gives way to the formation of memory B cells or long-lived plasma cells, which travel to bone marrow for the maintenance of immunological memory. Created by Biorender.

MZ-B cells can recognize T-independent antigens, which are transported by the blood, and can differentiate into short-lived plasma cells that secrete low-affinity IgM or initiate a response to antigens in a T-cell-dependent manner. FO-B cells, on the other hand, recirculate in the blood and in peripheral organs until they become activated by T-dependent antigens, and with the cooperation of T cells (T-follicular), these FO-B cells have the capacity to form germinal centers (GCs), where activated B cells (germinal center B cells, GC-B) use activation-induced cytidine deaminase (AID) to induce point mutations in the variable region of the B-cell receptor gene to generate a diverse population of GC B cells from the founder B-cell clone (somatic hypermutation, SHM) and class switch recombination (CSR) to enhance their affinity. The GC produces long-lived antibody-secreting plasma cells (ASCs) and memory B cells. Due to the random nature of this process, B cells with lower affinity, higher affinity, and novel autoreactivity are generated, necessitating strict selection of B cells with optimal antigen affinity to maintain tolerance and B cells with improved fitness (**Figure 1**).

Throughout this entire process of maturation and differentiation of B cells, self-reactive clones can be generated. To avoid this, there are central and peripheral tolerance mechanisms (32–35). Failure to eliminate autoreactive clones in conjunction with other factors, such as hormones, contributes to the development of autoimmune diseases.

In addition to B-2 B cells, B-1 cells are a unique subset of B cells that are distinct from conventional B-2 cells in terms of their development, phenotype, and function. B-1 B cells preferentially develop during fetal and neonatal life and maintain their peripheral presence into adulthood through antigen-driven self-renewal. These cells are innate-like B cells that are largely excluded from GC reactions and give rise to natural antibody-producing plasma cells (PCs) in a T-independent manner, including low-affinity-binding autoantibodies (36, 37). The secretion of autoantibodies has identified B-1 cells as potential contributors to the development of autoimmune diseases, such as lupus. In fact, elevated numbers of B-1 cells have been associated with autoimmunity both in human and mouse models (38).

## The role of hormones in preserving B-cell homeostasis

**Figure 2:**
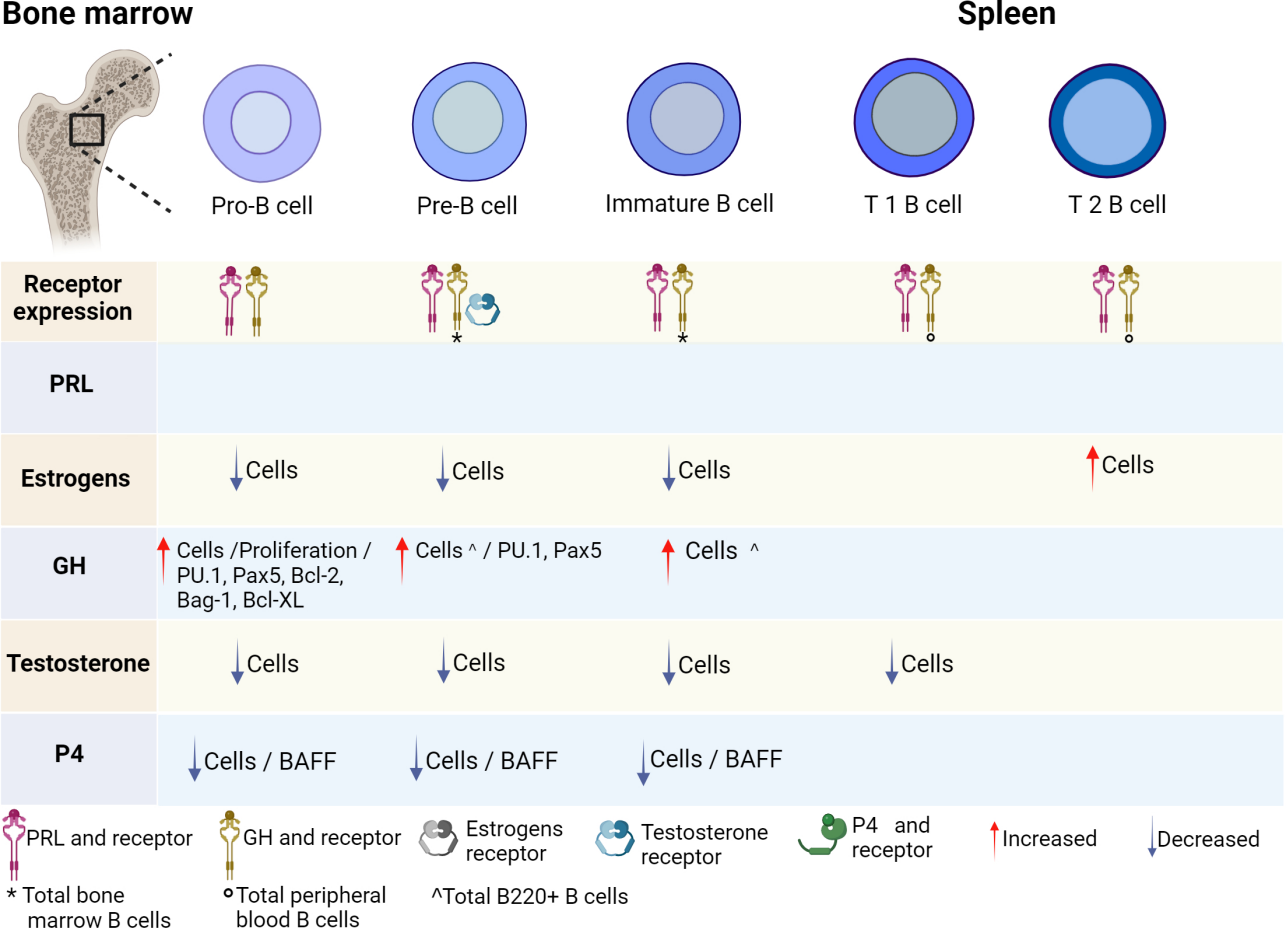
Hormonal effects on early B-cell development. B-cell development is regulated by hormones in the bone marrow. All stages of B-cell maturation express PRL and the GH receptor, as well as their ligands. Until now, only some stages of maturation have expressed testosterone receptor. Hormones positively or negatively regulate the differentiation of B cells and the expression of transcription factors, genes and cytokines that induce activation, apoptosis, or proliferation. PRL, prolactin; GH, growth hormone; P4, progesterone; BAFF, B-cell activating factor. Created by Biorender.

**Figure 3:**
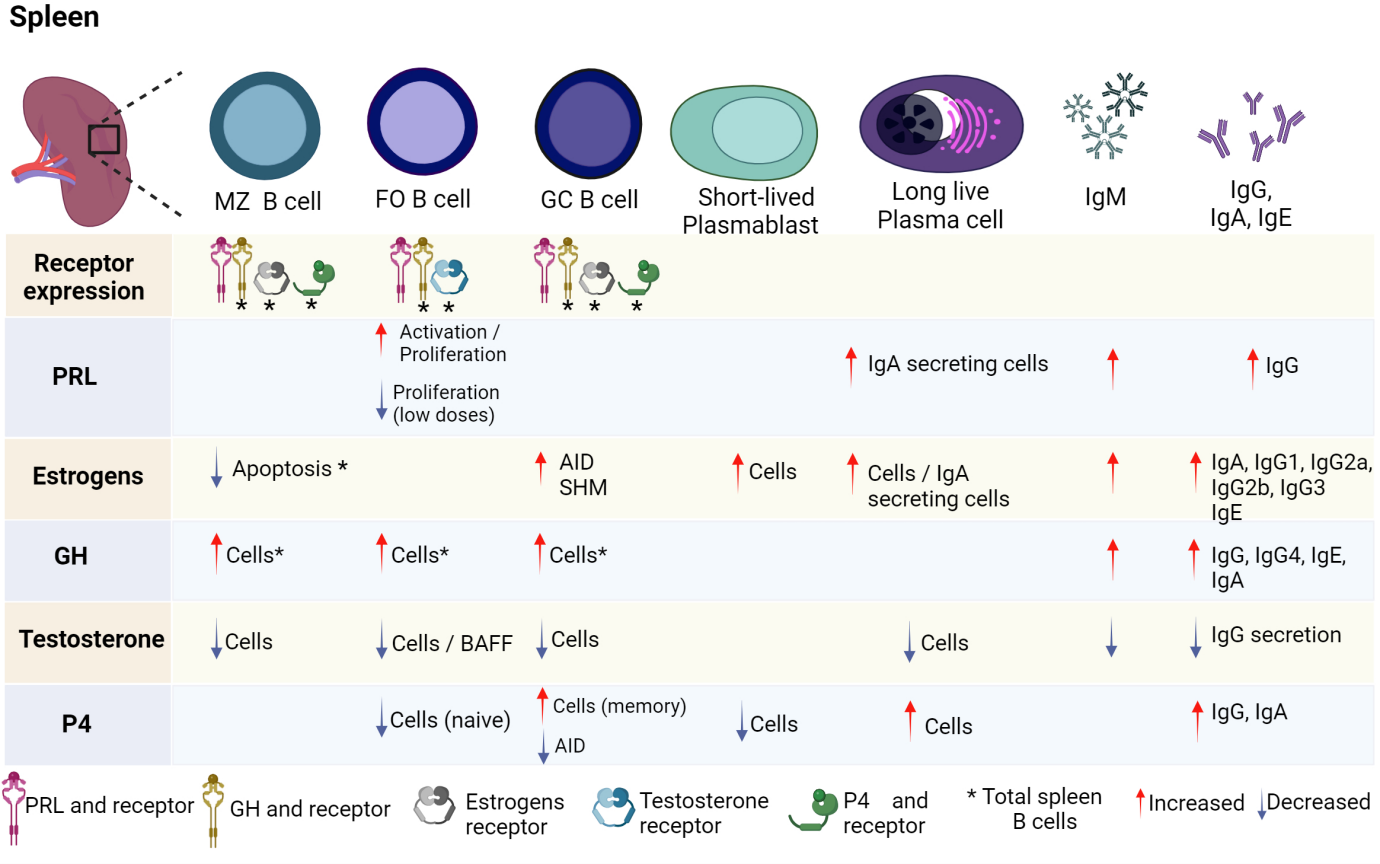
Hormonal effects on mature B-cell development. B-cell development is regulated by hormones in the spleen. FO, GC, and MZ B cells express PRL and GH receptor, as well as their ligands. Until now, only some stages of maturation have expressed estrogen, testosterone, and P4 receptors. Hormones positively or negatively regulate the differentiation of B cells and the expression of transcription factors, genes and cytokines that induce activation, apoptosis, or proliferation. PRL, prolactin; GH, growth hormone; P4, progesterone; BAFF, B-cell activating factor; SHM, somatic hypermutation; AID, activation-induced cytidine deaminase; FO B, follicular B cells; MZ B, marginal zone B cells; GC B, germinal center B cells. Created by Biorender.

Various studies have shown that hormones intervene in the development, activation, and proliferation of B cells. It has been reported that B cells can secrete different hormones (PRL, GH and P4) and can express different hormone receptors (PRL, estrogens, GH, testosterone, and P4) (**Figures 2**, **3**; **Table 1**).

**Table 1:** Receptor expression and synthesis of hormones in B cells.

Hormones	Receptor		Synthesis of hormone	References
	mRNA	Protein		
Prolactin	+	+	+	(39–42)
Estrogens	+ (ERα/ ERβ)	+ (ERα/ ERβ)	ND	(43, 44)
Growth Hormone	+	+	+	(45, 46)
Testosterone	+	+	ND	(43, 44)
Progesterone	+ (PR-A/PR-B)	+ (PR-A/PR-B)	+	(47–49)

+ Detected and reported in mice and human.

ND, not determined.

### Prolactin

PRL is a peptide hormone secreted by lactotroph cells of the anterior pituitary. Its monomeric isoform has a molecular weight of 23 kDa and is composed of 199 amino acids (50). Nearly 300 functions of PRL have been reported, and the development of mammary glands and lactogenesis appear to be the main functions of this hormone (51). Adenohypophyseal synthesis and secretion are essentially regulated at the hypothalamic level by substances such as dopamine, which exerts negative feedback on PRL (52). The biological effects of PRL are mediated by its interaction with its receptor, which depends on the type of cell where it is expressed. The PRL receptor belongs to the type I cytokine family and consists of three domains: the extracellular domain, which allows binding to the ligand; the transmembrane domain; and the intracellular domain. Various isoforms of the receptor have been described in both humans and mice (53, 54). PRL can also be produced by extrapituitary sites, which include B cells (55, 56). PRL production has been detected in IM-9-P cells, a human B-lymphoblastoid cell line, but the levels of PRL mRNA present in IM-9-P cells are lower than those found in the human pituitary. However, the IM-9-P PRL transcript is 150-200 nucleotides longer than its pituitary counterpart. Lymphoid-origin PRL is capable of carrying out an established biological function since it can stimulate the proliferation of Nb2 cells, which are dependent on lactogenic hormones such as PRL for cell growth (39).

According to Güneş & Mastro, the PRL receptor is also expressed in rat splenocytes, where it has been found at the mRNA and protein levels. Two isoforms (42 and 84 kDa) were found to be expressed throughout the estrus cycle. The 84 kDa isoform is expressed in greater abundance in proestrus, estrus, and diestrus, but both isoforms are expressed during pregnancy and to a greater extent during the beginning of lactation than in thymocytes (57).

The expression of the PRL receptor has also been found in human B cells, where its expression is similar in healthy individuals and in patients with hyperprolactinemia, even after treatment with bromocriptine, a dopamine agonist that regulates pituitary PRL production (58). In mice (C57BL, MRL, and MRL/lpr), the expression of the receptor at the mRNA and protein level has been reported in all stages of B cell maturation, both in bone marrow (pro-B, pre-B, and immature) and in the spleen transitional (T), FO, GC, and MZ B cells (40–42). In CAF1/J mice, treatment with PRL and estrogen increases the number of IgA-secreting plasma cells in mammary glands (59). *In vitro*, PRL (0.2-100 ng/mL) increases the activation of B cells, as measured by the increase in the expression of the IL-2 receptor (IL-2R) when the cells are costimulated with anti-IgM and IL-2, in addition to increasing the secretion of IgM and IgG (60). PRL also increases the proliferation of B cells when they are costimulated with suboptimal contractions of mitogens such as phytohemagglutinin (PHA) or *Staphylococcus aureus* Cowan 1 (SAC). However, compared to physiological concentrations (25 ng/mL), high concentrations of PRL (100 ng/mL) decrease B-cell proliferation (61). Furthermore, bromocriptine decreases the proliferation of B cells at DI-50 values between 1 and 10 µg/mL, as well as the production of immunoglobulins (62). In mouse B-cell hybridomas that produce IgM or IgG, PRL increases the proliferation and antibody levels of the hybridomas. In hybridomas 5C6, 4A8, 19B3, 4D8, 3B10, and 4A2, proliferation increased at concentrations ranging from 1-50 µg/mL. Furthermore, the effect is increased when cells are stimulated with IL-4, IL-5, and IL-6. PRL (1-50 µg/mL) also restored the proliferation of hybridomas 5C6, 7A8, 3C9, 4D8, and 3B10 upon inhibition induced by TGF-β (63). The PRL receptor is expressed in all maturation states of B cells, indicating that this hormone could play a role in the development of B cells in the BM. Meanwhile, in mature B cells, PRL at physiological concentrations could participate in the activation, proliferation, and differentiation to ASC, thus increasing the production of antibodies, if the B cells have a co-stimulatory signal (anti-IgM, mitogens, etc.); it could also act in an autocrine manner since B cells can secrete PRL. In contrast, high-dose PRL reduces the proliferation of B cells, possibly through the suppressors of cytokine signaling (SOCS) pathway, as it has been reported that the PRL induces SOCS 1 and 3 in a dose-dependent manner (64).

On the other hand, an increase in the number of circulating B cells has been reported in healthy men when they perform physical exercise. Additionally, the serum concentration of PRL was increased in these men, which correlated positively with the number of B cells that express the PRL receptor, and the number of B cells increased with exercise (65). However, in B cells from healthy subjects and hyperprolactinemic patients, PRL has been reported to have no effect on the *in vitro* expression of the costimulatory molecules CD40 and CD86 (66). These results suggest that in humans, PRL could participate in the proliferation of B cells but not in their activation.

In general, these results show the immunomodulatory effects of PRL on the development, activation, proliferation, and differentiation of B cells, indicating that PRL plays an important role in communication between the endocrine system and immune system.

### Estrogens

Estrogens are hormones derived from cholesterol and are associated with female reproductive organs and the development of primary and secondary sexual characteristics. These hormones regulate the physiological processes of germ cell maturation and fertilization preparation (67, 68). Estrogens included estrone (E1), estradiol (E2, 17β-estradiol), estriol (E3), and estetrol (E4). Estradiol is the predominant circulating hormone among the estrogen group. In contrast to estetrol, which is produced only by the fetal liver, estrone and estriol are produced mainly by granulosa cells and ovaries but can also be secreted by the adrenal glands, adipose tissue, and cells of the immune system, including B cells (69–71).

Two types of intracellular estrogen receptors have been described, estrogen receptor α (ERα) and estrogen receptor β (ERβ), both of which are distributed in the immune system. The estrogen receptor has been found to be expressed in B-cell lines (U266 and RPM1 8226 cells line) (43). In the C57BL/10 mouse strain, it has been reported that splenic B cells express ERα both *in vivo* and *in vitro* after stimulation with 100 nM 17β-estradiol (E2), but they do not express ERβ (44). In the spleen B cells of BALB/c mice, the expression of ERα and ERβ was demonstrated (72), and in the same mouse strain stimulated *in vitro* with estrogen (1 nM), the expression of activation-induced deaminase (AID) mRNA increased (73). Furthermore, ERα binds upstream to the human AID promoter both *in vivo* and *in vitro*. Estrogens also induce *in vitro* increases in IgA, IgG1, IgG3 and IgE levels in mouse spleen B cells; increase the frequency of mutations in the VH and CD95/Fas loci of the Burkitt lymphoma cell line (Ramos); and cause mutations in the Sγ3 region of spleen cells from the intersection of AID^−/−^ and BALB/c mice (73). Therefore, estrogens play an important role in AID-dependent processes, such as isotype switching and somatic hypermutation (SHM). AID can be activated by estrogen, which can result in immune hyperstimulation. This activation can occur directly when the estrogen receptor (ER) binds to the AID promoter or indirectly through the activation of transcription factors that boost AID expression (e.g., HoxC4) (74).

In B-cell cultures from men and women treated for 6 days with pokeweed mitogen (PWM) and estradiol (26,000-260 pmol/L), an increase in the number of plasmablasts and IgM-secreting plasma cells has been observed (75). In mice that expressed ERα^+/+^, estradiol treatment (5 mg/kg), accelerated the maturation of B cells by increasing the number of mature B cells and decreasing the number of pre-B cells and immature B cells (76). These results were verified by Erlandsson et al., who treated male mice that expressed ERα^+/+^ or ERβ ^+/+^ with estradiol (30 µg/kg) and found that estradiol inhibits the lymphopoiesis of B cells and increases the secretion of immunoglobulins (77). Therefore, estrogens decrease lymphopoiesis at the pro-B-cell stage (78).

Estrogens were found to increase the percentage of IgG- and IgA-secreting cells in rhesus macaques; this change is also related to the menstrual cycle and represents a link between ovarian hormones and the development of B cells (79). In the BALB/c mouse strain, *in vitro* estrogens were shown to protect splenic B cells from apoptosis and increase IgG secretion. However, they did not promote B-cell differentiation into plasma cells or proliferation (80).

According to these studies, B cells have estrogen receptors. Estrogens have been observed to reduce the population of B cells in the BM. Conversely, in the spleen, estrogens increase AID-dependent processes, such as isotype switching and SHM, and it increases Ig secretion while preventing B cells from undergoing apoptosis. Therefore, it is likely that estrogens act mainly in mature B-cells and play an important role in their differentiation, activation, and survival.

The aromatization of androgens is a key step in estrogen production, and the aromatase enzyme converts androgen to estrogen. In transgenic male mice that express human aromatase (AROM^+^), which has a high estrogen/androgen ratio, alterations in the function of B cells have been identified. AROM^+^ mice presented higher concentrations of IgE, similar to the physiological concentrations in females, contrary to what was observed in mice with knockout (KO) of the aromatase enzyme (ArKO), characterized by an imbalance in sex hormone metabolism, resulting in nondetectable levels of estrogen in serum and elevated levels of circulating gonadotrophins and testosterone (81). Furthermore, high circulating levels of estrogen in mice increase the production of IgG1, IgG2a, IgG2b, IgG3, and IgA antibodies, as well as anti-DNA antibodies, which are involved in autoimmune diseases, such as SLE. An increase in the number of mature B cells (CD19^+^IgD^low^, IgM^high^) and plasma cells (CD19^+^CD138^+^IgD^−^) that contribute to the production of immunoglobulins has been reported (82). Additionally, 244 of 362 transcripts were altered in B cells due to estrogen/androgen imbalance. Therefore, the synergistic and disruptive effects of sex hormones such as estrogens directly contribute to the development of autoimmune diseases by altering the differentiation of B cells (82).

Thus, in addition to their involvement in numerous other functions, estrogens play a vital role in metabolism, cognition, and immunity. Moreover, elevated androgen and estrogen levels cause alterations in B-cell development and Ig production.

### Growth hormone

GH is produced through the anterior pituitary in acidophilic somatotroph cells. Its synthesis and release is regulated by growth hormone-releasing hormone (GHRH), which is secreted by the hypothalamus and by ghrelin. At the same time, it is negatively regulated by somatostatin (GHIH). GH modulates its biological functions through a receptor member of the I cytokine receptor family. GH is necessary for the postnatal development as well as for bone and muscle development (83, 84).

In human peripheral B cells from healthy subjects, the expression of GH and its mRNA receptor has been determined (45). Additionally, an age-related increase in GH receptor expression has been reported in B cells (85). Moreover, in B-cell lines (Raji and Daudi cell lines), GH and its mRNA receptor was found (45). In C57BL/6, BALB/c and DBA/2 mice, the expression of the GH receptor has been detected in BM (pro-B, pre-B, immature cells), peripheral B cells, and spleen (46) (**Figures 2**, **3**).

In the pro-B-cell line (Ba/F3), which is dependent on IL-3, transfection with the GH receptor induces cell proliferation (86). Meanwhile, GH allows the cell cycle to progress from the S to the M phase, promoting the expression of E and A cyclins, c-myc and inhibiting the expression of p27, while maintaining the constant expression of Bcl-2, Bag-1, and Bcl-XL (antiapoptotic proteins) (87, 88). GH induces different signaling pathways in these pro-B cells and can activate the NF-κB signaling pathway to exert an antiapoptotic effect; also induces PI3-K signaling, which is responsible for a proliferative effect that can be regulated by c-myc and cyclins (87, 88). Since GH can activate the NF-κB signaling pathway, it is likely that it also exerts an inflammatory effect through the production of cytokines and chemokines, such as TNF-α, IL-6, and MCP-1, as it has been reported in preadipocytes (89). These cytokines are critical for immune cell development, differentiation, and regulation. Therefore, the balance between the pro-inflammatory and immunosuppressive functions of these cytokines and their implications for the pathogenesis of autoimmune diseases are critical.

Similarly, GH promotes the phosphorylation of STAT5b and its eventual translocation to the nucleus, where it binds to the proximal STAT response element (pSRE) in the SOCS3 promoter, which regulates the response to cytokines. Notably, the expression of SOCS3 is aided by the activation and recruitment of JNK, p38 MAPK, c-Fos, c-Jun, and FOXO3a, which are associated with the AP1/CRE and FOXO binding motifs on the cAMP response element-binding promoter (90). However, in C57BL/6 mice, the percentage of B220^+^ cells were increased after BM cells were cultured in the presence of exogenous GH. Exogenous GH also increased the mRNA expression of Ig-α/CD79a and Ig-β/CD79b, which are important for BCR signaling, and the mRNA expression of PU.1 and PAX5, which are important for early B-cell development (91).

The absence of GH in GH-releasing hormone knockout mice induces a decrease in body weight and the percentage of spleen B cells. However, the percentage of B cells normalizes during aging (92). In GH-deficient children who are administered biosynthetic GH, the percentage of B cells tends to decrease but the effect is transient and normalizes over a period of 9 months (93). Moreover, GH increases the *in vitro* levels of IgG, IgG4, IgE, IgA, and IgM antibodies in the B cells of healthy subjects (94, 95). Hence, GH is important for B cell interactions since GH is involved in the control of the development, maturation, and the number of B cells in the periphery.

GH is a crucial regulatory protein in numerous B cell processes, including signal transduction, checkpoint regulation, apoptosis, cytokine production, and cell division. Furthermore, understanding the role of B cells in autoimmune diseases and the subsequent production of pathogenic autoantibodies enables us to understand how hormonal regulation of B cell-intrinsic signals (ontogeny and function) may contribute to autoimmunity pathogenesis. This underscores the crucial role of B cells in the interconnected communication systems of the immune and endocrine systems.

### Testosterone

Testosterone is the main sex hormone in men and the most common androgen in adult men. Testosterone is secreted in the gonads and adrenal cortex, mainly in the Leydig cells of the testes (96); it is responsible for regulating different physiological processes, including secondary sexual characteristics associated with puberty, muscle hypertrophy, erythropoiesis, bone metabolism, cognitive functions, and mood (97). In men, the free fraction of testosterone is 2%. Testosterone binds to sex hormone binding globulin (SHBG) with high affinity and albumin and other proteins with lower affinity, which impacts its distribution in the blood circulation (98). It has been reported that splenic B cells from C57BL/6 mice express the intracellular androgen receptor (iAR), which is located in the cytoplasm and can translocate to the nucleus after stimulation with testosterone (44) (**Figure 3**). Furthermore, the expression of the androgen receptor has also been determined in the ReH-6 (early pre-B) and RAJI (pre-B) cell lines (43) (**Figure 2**).

Since the 1970s, testosterone has been known to negatively regulate lymphocyte development. In female C3H/He mice that were subjected to irradiation and treated with syngeneic bone marrow cells, there was a reduction in the number and size of splenic follicles and a decrease in the number of lymphocytes surrounding the GC and plasma cells when treated with testosterone (1-20 mg/mL); the decrease in plasma cells was maintained until 60 days posttreatment (99). In addition, B-cell lymphopoiesis in the BM and spleen is reportedly increased in castrated mice (100–102). In C57BL/6 castrated mice, treatment with testosterone or 5α-dihydrotestosterone decreased the number of BM B cells (103). Chen et al. reported that castrated rats with low endogenous testosterone levels develop splenomegaly, while the supply of exogenous testosterone restores the size of the spleen, similar to physiological conditions (104). In addition, LPS stimulation combined with low testosterone levels increases the *in vitro* production of nitric oxide and TNF-α and the proliferation of splenocytes, and TNF-α production negatively correlates with plasma testosterone concentrations (104). Therefore, homeostatic concentrations of testosterone may regulate the development, proliferation, and maturation of B cells and have an anti-inflammatory effect.

It is probable that testosterone interferes during the early stages of B-cell development, since in castrated C57BL/6 mice, the number of newly migrated B cells from the BM (B220^low^CD24^high^) increases, as does the number of early-stage pro-B cells in the BM (101). Testosterone has an indirect effect on B-cell lymphopoiesis by acting on marrow stromal cells, where it increases the mRNA expression of TGF-β (105). Wilhelmson et al. reported that in male androgen receptor KO mice, the osteoblast lineage cell-specific receptor KO (O-ARKO) allele was associated with increased expression of B-cell precursors, pro-B cells, large pre-B cells, small pre-B cells, and immature B cells (106). Therefore, androgens such as testosterone, through cells of the osteoblast lineage, exert inhibitory effects on the lymphopoiesis of BM B cells.

In both men and women, *in vitro* culture of PBMCs and purified B cells with testosterone (100 nM) for 7 days inhibited the production of IgG and IgM, while the addition of IL-6 in culture partially restored the production of these antibodies in PBMCs treated with testosterone. However, testosterone affects the production of IL-6 by monocytes but not by T and B cells (107). Therefore, this hormone likely induces an indirect effect on the inhibition of immunoglobulins through monocytes.

Among the inductive mechanisms that establish the decrease in B-cell expansion by androgens, testosterone has been shown to regulate B-cell tolerance since the loss of the androgen receptor induces B-cell evasion of apoptosis (108). In B-cell CD19+ androgen receptor knockout (G-ARKO) mice, this deficiency increases the number of splenic B cells, while the addition of exogenous testosterone *in vitro* (25 μg/day) decreases the number of splenic B cells only in castrated control mice. Additionally, testosterone decreases the number of splenic B cells at different stages of maturation, including B1, transitional (T1, T2 and T3), FO and MZ cells (109). Furthermore, testosterone downregulates BAFF expression in splenocytes in an androgen receptor-dependent manner. It is important to mention that the serum levels of this hormone are notably greater in females and in men with hypogonadism (109), which is consistent with previous reports on the predominance of autoimmunity in females. Thus, testosterone controls many physiological functions, such as immunity, primarily through directly influencing B cells that express androgen receptors. Therefore, testosterone plays a protective role against the development of autoimmune diseases.

### Progesterone

P4 is synthesized by ovarian follicles through the regulation of theca cells and granulosa cells. P4 plays an important maintenance role in mammalian pregnancy and fetal development (110). The biological action of P4 depends on two intracellular receptors, PR-A and PR-B (111). Many of the effects of P4 are related to the inhibition of the immune system, particularly the inflammatory response, which involves a large number of proinflammatory cytokines (112, 113).

It is worth mentioning that hormonal concentrations fluctuate throughout life since they increase during puberty, change cyclically during the menstrual cycle, and increase again during pregnancy. Therefore, revealing the general hormonal regulation of the immune system by merely observing physiological effects is difficult since, for example, circulating P4 levels in male mice range from 1.5–2 ng/mL, while those in females range from 3 to 35 ng/mL (114–116). However, during pregnancy, P4 levels are exacerbated (250 mg during the third trimester) and decrease before childbirth. In this physiological state, P4 is mainly immunosuppressive (113, 117).

The expression of the P4 receptor (protein and mRNA) has been demonstrated in the bursa of Fabricius and the thymus in chickens (49). In mice, mouse (BALB/c) splenic B cells express PR-A and PR-B, with PR-A predominating in these cells (47). Both isoforms have also been found in the bursa of Fabricius cells following estradiol treatment (49). Furthermore, it has been suggested that B cells produce P4 since the presence of P4 has been detected in the supernatant of rat B cells activated with concanavalin A (48, 118) (**Figures 2**, **3**). The expression of progesterone and its receptor in B cells suggests that this hormone may act as an autocrine manner in these cells.

During pregnancy in mice, a gradual decrease in pro-B, pre-B and immature cells in the BM, as well as in the concentration of BAFF, has been reported (119). In male lymphocytes, P4 induces a significant downregulation of BAFF (120). The results suggest that P4 tends to have protective and regulatory effects on the immune response during pregnancy by decreasing the concentration of BAFF, which could cause a decrease in B cell maturation. P4 increased the apoptosis of different B cell lines (Raji, CRL 1596 and SKW) *in vitro* (121). In BALB/c mice, P4 inhibited the activation of B cells *in vitro* by decreasing the expression of costimulatory molecules, such as CD80 and CD86 (122). This decreased the presentation of antigens and, therefore, the activation of B cells, which favors the apoptosis of cells that do not activate or proliferate. Therefore, P4 tends to regulate B cell maturation and activation and may be an important regulator of tolerance in B cells.

P4 increases the number of IgA plasma cells in the uterus of ovariectomized outbred albino mice *in vivo* (123). While in B cells from spleen of BALB/c mice, P4 did not increase IgG production (122), nor in the coculture of follicular T cells/B cells from healthy women and activated with 1 μg/mL staphylococcal enterotoxin B (SEB); the addition of a pregnancy-related dose of P4 (50 ng/mL), P4 did not increase the IgG production (124). The increase in IgA may be due to the microenvironment of the uterus since other hormones could favor this increase. Furthermore, in splenic B cells from BALB/c mice, the expression of AID mRNA was reduced *in vitro* in the presence of P4 (73); therefore, it could induce a decrease in isotype switching.

P4 also regulated plasma cell differentiation since it downregulated the expression of CD138 (122), and reduced the percentage of plasmablasts (CD19+ CD38+ CD138-), but increased the percentage of plasma cells (CD19+ CD38+ CD138+) in coculture of follicular T cells/B cells (124), which suggests that the effects of P4 also depend on the maturation state of the B cells. Additionally, P4 increases the percentage of IL-10^+^ follicular regulatory T cells (124); therefore, P4 modulates the activation of B cells and could regulate GC response during plasma cell generation.

## The role of hormones in autoimmune diseases

**Figure 4:**
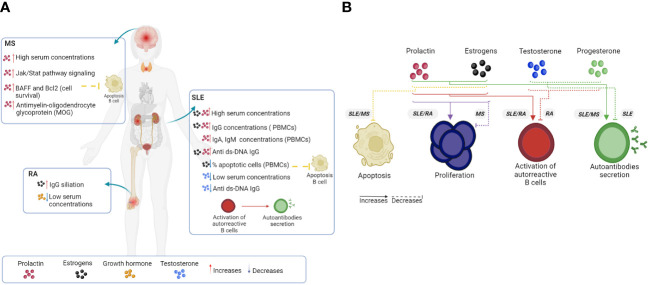
Hormonal effects on B cells and autoimmune disease. The loss of a homeostatic physiological state, such as the set of anomalies of the immune system (loss of tolerance) and of some of the hormonal elements, increases the hyperactivation, proliferation and differentiation of B cells, as well as the expression levels of different proteins that promote the survival of autoreactive clones that produce autoantibodies. In human **(A)** and mouse **(B)** models, hormones such as PRL, GH and estrogens generally favor an increase in the clinical manifestations of systemic autoimmune diseases such as SLE and RA, as well as the severity of organ-specific diseases such as MS. However, a decrease in testosterone, P4 and regulatory cellular elements is injurious in the control of pathologies. AR, Rheumatoid arthritis; SLE, Systemic lupus erythematosus; MS, Multiple sclerosis. Created by Biorender.

It has been documented in animal models and human clinical studies that alterations in hormonal homeostasis contribute to the development of autoimmune diseases. The effects of hormones on B-cell function and vice versa can promote the development of autoimmune diseases such as SLE, RA, and MS (**Figures 4A, B**).

### Systemic lupus erythematosus

SLE is a chronic inflammatory autoimmune disease caused by the hyperactivity of B cells, which is identified by the presence of autoantibodies directed against various molecules in the nucleus, such as DNA, RNA, Ro, La, and histones. These autoantibodies can also form immune complexes that frequently leave circulation and are deposited in the kidney, skin, and brain, among other tissues, where they can cause inflammation and tissue damage. SLE affects mainly women of reproductive age at a 9:1 ratio with respect to men (125, 126).

The increased incidence of lupus in women of reproductive age points to a hormonal influence on the development of the disease. Sex hormones, such as estrogen and PRL, have been found to exert immunostimulatory effects on B cells that induce the development of autoimmunity. In humans, PRL increases the production of IgA, IgM, and IgG in the PBMCs of healthy individuals and, to a greater extent, in patients with SLE, as well as the synthesis of anti-dsDNA IgG *in vitro* (127, 128). Furthermore, between 10 and 33% of patients present with hyperprolactinemia, which is correlated with disease activity (129, 130). Moreover, estrogen also increases the secretion of total IgG in patients with SLE (active and inactive) and of anti-dsDNA IgG (active SLE) (131). Estrogens even decrease the percentage of apoptotic cells in PBMCs from patients with SLE and healthy individuals (132) (**Figure 4A**).

Similarly, in the NZB/W F1 mouse strain, which is genetically predisposed to develop an SLE-like disease, estrogen-induced hyperprolactinemia is associated with premature death (133). Even in MRL/Fas^lpr/lpr^ mice, which also develop SLE-like disease, estrogens increase immune complex-mediated glomerulonephritis (IgG1, IgG2a, IgG3, IgM; anti-dsDNA), lymphoproliferation, and mortality (134–136). Similarly, in MRL and MRL/Fas^lpr/lpr^ mice, hyperprolactinemia induced by the drug metoclopramide increases the production of anti-dsDNA antibodies, proteinuria and the expression of the PRL receptor in transitional B cells (41). Therefore, estrogens and PRL modulate the maturation of autoreactive B cells.

There is a whole range of mouse model studies in which PRL, and estrogens were identified as modulators of B-cell tolerance, activation, proliferation, and maturation (**Figure 4B**). Regarding tolerance mechanisms, in BALB/c transgenic mice harboring the H chain of an anti-DNA Ab (R4A-γ2b), estrogens alter B-cell tolerance and increase the number of splenocytes through the positive regulation of the antiapoptotic Bcl-2 protein, which is associated with the evasion of autoreactive B-cell apoptosis (137). Similarly, PRL also increases the expression of Bcl-2 in the splenic B cells of R4A-γ2b mice (138). In MRL and MRL/Fas^lpr/lpr^ mice, elevated levels of PRL decrease the absolute numbers of pro-B and immature cells in both strains and increase the expression of the antiapoptotic molecule BIRC5 (mRNA) in pro-B (MRL/Fas^lpr/lpr^) and immature cells (MRL and MRL/Fas^lpr/lpr^) (42). PRL promotes immature B-cell survival through the activation of STAT3, which binds to the promoters of the antiapoptotic Birc5, Bcl2a1a and Bcl2l2 genes (139, 140). Therefore, the number of T1 B cells increases. Moreover, in R4A-γ2b mice, estrogens and PRL increase the expression of BAFF in T1 B cells (138, 141). Additionally, in Sle3/5 R4A-γ2b C57BL/6 mice that express both the H chain transgene and the Sle3/5 lupus susceptibility range, PRL decreases the T1:T2 B ratio and promotes autoreactive B-cell accumulation in splenic follicles. Moreover, PRL increases the expression of CD40, B7-2, and MHC II in B cells; CD40-L in T cells; and B7-2 and CD44 in dendritic cells and monocytes, through which PRL can be activated directly and indirectly from dendritic cells to B cells (142, 143). Similarly, PRL increased the expression of CD40 in the splenic B cells of R4A-γ2b mice (138). However, in C57BL/6 mice harboring the Sle1 lupus susceptibility allele, ERα signaling increases in females, favoring the activation of B cells through an increase in B220^+^CD86^+^ and B220^+^CD22^+^ cells (144). Therefore, ERα signaling is differentially activated in females with SLE through Sle1b. Taken together, these data confirm that PRL and estrogens promote the hyperactivity and survival of B cells through genetic and immunoregulatory factors during the progression of SLE.

Furthermore, the hormonal microenvironment contributes to choosing the fate of transgenic R4A autoreactive B cells since estrogen favors the formation of MZ B cells, and PRL favors the development of FO B cells. Although estrogens also influence BCR signaling by decreasing calcium flux in B T1 and B T2 cells (141), estrogens and PRL also regulate antibody duration. The concentration of anti-DNA antibodies persists for at least 12 weeks after withdrawal of the estrogen stimulus, as do the number of DNA-reactive splenic B cells, immune complex deposition, and survival of the MZ-B cells. However, a constant effect of PRL is necessary to maintain anti-DNA reactivity in serum and FO-B cells (145). Therefore, the estrogen-susceptible survival of MZ autoreactive B cells is an important source of autoantibodies during disease progression.

In mature B cells, PRL and estrogens influence the GC reaction. In MRL/Fas^lpr/lpr^ mice, PRL increases the proliferation of GC-B cells, the differentiation of antibody-secreting cells, and the production of IgG3-conjugated anti-dsDNA antibodies. PRL signals in GC-B cells through the long isoform of its receptor via STAT1 and AKT (40). In C57BL/6 Sle1 mice, ERα signaling increases in females, favoring the formation of spontaneous germinal centers (GCs) and the development of anti-chromatin antibodies of the IgG isotype (144). Therefore, PRL and estrogens influence the maturation and proliferation of B cells, favoring the maturation of autoreactive clones capable of differentiating into antibody-secreting cells (ASCs) and increasing the manifestations of SLE through immune complexes.

Epigenetically, estrogens (ERαs) reverse the protection of the MRL/Fas^lpr/lpr^ strain mediated by histone deacetylase inhibitors (HDIs) and short-chain fatty acids (SCFAs) *in vivo*, which reduce skin lesions and the production of anti-dsDNA IgG2a. *In vitro*, ERα reverses the negative regulatory effects of HDIs on the expression of the AID gene and Aicda gene and on class switch DNA recombination (CSR) through the negative regulation of miR-26a, which is expressed in activated B cells that carry out CSR and plasma cell differentiation (146). On the other hand, in BWF1 mice, which spontaneously develop lupus, chronic administration of 17β-estradiol (E2) increased the secretion of anti-DNA and anti-Br-RBC IgM autoantibodies in B1 cells *in vitro* (147), highlighting the importance of this population in autoimmunity.

There are pharmacological strategies that aim to reduce the manifestations of SLE. In NZB/W F1 mice treated for 5 months with tamoxifen (TAM), a synthetic antiestrogen, a decrease in cellular infiltration (CD40^+^ and CD40L^+^ cells) and glomerulonephritis mediated by estrogens were reported at 8 months of age (148). Moreover, simultaneous treatment with bromocriptine, a dopamine agonist, reduces manifestations of SLE (induced by PRL or estrogen), such as anti-DNA antibody production and mortality (149–151).

In parallel, compared with male patients, female patients with active and inactive SLE exhibit an increase in serum estrogen levels and a decrease in the levels of testosterone and its precursor dehydroepiandrosterone (DHEA-S) (152). It has been determined that testosterone (10^-^9 M) suppresses the production of IgG and IgG anti-dsDNA antibodies in the PBMCs and B cells of SLE patients *in vitro* (153). However, in NZB/W F1 mice, testosterone promoted the development of the Gr^high^Ly-6G^+^CD11b^+^ myeloid population, which inhibited differentiation into ASCs *in vitro*. Furthermore, the depletion of Gr1^+^ cells *in vivo* increases the serum production of antinuclear antibodies (anti-dsDNA and anti-histone) in male mice but has no effect in females (154). An *in vivo* decrease in Gr1*+* cells favors the formation of GCs and the number of GC-B cells (155). With respect to the P4 receptor, Nba2 KO mice exhibit an increase in IgG1 and IgG2c serum antibodies, an increase in immune complexes, and a decrease in survival (156). Together, testosterone and P4 play therapeutic roles in SLE by suppressing the secretion of antibodies from B cells and their progressive damage (**Figures 4A, B**).

### Rheumatoid arthritis

RA is a chronic autoimmune disease that affects the joints. RA is characterized by inflammation of the synovial membrane and the formation of a pannus, an abnormal set of granulomatous tissue that is responsible for the destruction of cartilage and bone as a consequence of the activation and infiltration of cells of the immune system, including T and B cells (157). RA affects 1% of the world's population, with a 3:1 female/male ratio. RA patients exhibit an increase in autoantibodies against IgG, known as rheumatoid factor (RF), and anti-cyclic citrullinated peptide antibodies (ACPAs) (158, 159).

Several genes related to the immune response (TLR7, CD40L, FOXP3, etc.) have been reported in an RA murine model (collagen-induced arthritis [CIA]), and genes expressed on the X chromosome were shown to regulate susceptibility to RA (160, 161). Since RA is more prevalent in women, it has been proposed that it may also be regulated by hormonal factors. However, there are controversies about the influence that estrogens can have on the development of RA. Several studies have suggested that estrogen decreases susceptibility to developing RA. Using mice susceptible to developing RA (DBA/1 strain), Nilsson et al. reported that mice are more susceptible to developing this disease when they are ovariectomized and treated with low doses of antigen (CII). Moreover, the number and percentage of B220^+^CR1^+^ (complement receptor 1) B cells in the spleen, lymph nodes, and peripheral blood, as well as the expression of CR1 were decreased in these mice; therefore, estrogens could increase the expression of CR1 (162). The protective effect depends on the dose of CII because CR1 can inhibit inflammation only when mice are triggered by low antigen (CII) doses but can stimulate an immune response with high antigen doses. Therefore, the protective effect of estrogens depends on the dose of the antigen. Moreover, combined treatment with dexamethasone plus estrogen in these same mice (ovariectomized DBA/1) decreased the frequency of B cells, the concentration of autoantibodies, and the incidence of inflammation (IL6), reducing the manifestations of the disease, destruction of cartilage, and osteoporosis. The same results were obtained with dexamethasone plus raloxifene, a selective estrogen receptor modulator (SERM) (163). It is known that in RA, the presence of autoantibodies precedes the inflammatory phase; the sialylation state of these antibodies is important in this disease. Low IgG sialylation has been related to the progression of inflammation, while high IgG sialylation decreases the manifestations of the disease (164). It has been reported that high levels of estrogen increase the expression of ST6GAL1, the enzyme responsible for binding sialic acid to IgG, thus increasing the sialylation of IgG and probably inducing an anti-inflammatory effect in patients with AR (165). Therefore, estrogen may have a protective effect on RA by reducing inflammation. By increasing IgG sialylation, the expression of CR1 in B cells decreases, as does the expression of IL6.

However, it has also been reported that estrogen promotes the development of RA. Estrogens increase the expression of HLA-DR4 in B cells from DRB1*0401^-^CIA transgenic male mice (mice that develop RA and produce RF and ACPAs) and increase the antigen-specific response to estrogen CII peptides (residues 254-273) restricted to DR4. Furthermore, HLA-DR4 has been associated with the presence of RA in both female patients and mice. DRB1*0401 females are more susceptible to developing CIA, with increased concentrations of proinflammatory cytokines (IL-2, IL-4, IL-5, and IL-13) and increased proliferation of B cells. Similarly, the presentation of citrullinated peptides by B cells is more efficient in females than in males. During *in vitro* antigen presentation, female B cells tend to produce higher concentrations of IL-13 and IL-4, while males produce higher concentrations of IL-10, IL-1β, and TNF. Simultaneously, males express a greater number of regulatory B cells (CD5^+^CD1^hghi^) (166). Therefore, sex differences in RA include immunoregulation of estrogens and androgens in the B-cell response and differential expression of DR4, as well as the regulatory cells that influence the development of autoimmunity.

Therefore, estrogen may have a protective effect by reducing inflammation. By increasing IgG sialylation, the expression of CR1 in B cells decreases, as does the expression of IL6. However, it could favor the development of RA when B cells express DRB1*0401 (associated with the presentation of autoantigens); estrogens increase the expression of DRB1*0401, increasing the presentation of autoantigens and, therefore, the presence of autoantibodies. These findings show that estrogens may have a dual role in B cell immunomodulation. However, the dose appears to be a crucial factor in predisposition or susceptibility to RA, explaining the marked prevalence based on gender.

In the same CIA model, it has also been reported that mice with deletion of a P4 receptor allele in osteoprogenitor cells (PR ΔPrx1-CIA) have a greater incidence of osteoarthritis. Male mice have lower bone mass in the legs and knees, a lower volume of subchondral trabecular bone and greater erosion and damage to the cartilage of the knee joint (167). Therefore, P4 plays a protective role in the manifestations of RA. Similarly, GH concentrations are deficient in patients with RA (168).

Although hormonal imbalances can have direct effects on RA, B cells play an interesting role in the development of pathology (**Figures 4A, B**). Thus, maintaining estrogens, P4 and GH levels at physiological levels would aid in preventing the disease in patients.

### Multiple sclerosis

MS is an inflammatory autoimmune disease that affects the central nervous system and is characterized by chronic demyelination, axonal loss, and eventual neuronal degeneration (169). It most frequently affects women between 20 and 40 years old, with a female/male ratio of 4:1, depending on geographic location (170, 171). MS affects a total of 2.8 million people worldwide, and in recent years, the number of MS cases has continued to increase, with a high incidence in women (170). Although MS is mediated mainly by T cells, B cells significantly contribute to this disease (172).

There is evidence suggesting the involvement of hormones in the course of MS, although it is controversial whether the immunoregulatory effects are protective or detrimental to the disease. For example, in MS patients receiving infertility treatment with assisted reproductive technology, in which there is an increase in the serum levels of 17β-estradiol and P4, MS activity increases 7-fold (173). On the other hand, it has also been reported that the relapse rate of MS decreases during pregnancy and increases during the first postpartum trimester (174). Therefore, although the study suggested that a systemic hormonal increase can confer immunosuppressive effects, there is no insight into the mechanisms through which the hormones could exert their effects.

Estrogens have been shown to slow the development of experimental autoimmune encephalomyelitis (EAE) in female C56BL/6 mice that underwent MS modelling with pertussis toxin. In mice with EAE, prolonged treatment with high doses of estrogen increased the percentage of IL-10-producing CD1d^high^CD5^+^ regulatory B cells (175). It was proven that estrogens exert their protective effect on B cells; for example, in B-cell-deficient mice (μMT-/-), the protective effect was lost (176). It has been reported that the interaction of estrogens in B cells occurs through ERα and G protein-coupled receptor 30 (GPR30) (177).

When B cells from female C57BL/6 mice were preincubated with low doses of estrogens and subsequently coincubated with myelin oligodendrocyte glycoprotein (MOG) 35–55-specific 2D2 TCR Tg T cells, the proliferation of MOG35–55-specific CD4+ T cells decreased compared with that in B cells not preincubated with estrogens; this could be explained by the fact that estrogens increase the expression of PDL1 (immune response inhibitory molecule) (176).

It has been reported that during pregnancy, in addition to hormonal changes, there are changes in the intestinal microbiota (178). Therefore, hormones could also induce changes at the microbiota level. Chronic estrogen treatment has been shown to positively regulate the microbiota of C57BL/6-EAE mice and increase the frequency of CD19^+^CD5^+^CD1d^high^ regulatory B cells in the spinal cord and mesenteric lymph nodes. Thus, estrogens have a protective effect against EAE and its clinical manifestations, such as dysbiosis, through increasing the percentage of regulatory B cells and enriching a favorable microbiome (179). Furthermore, estrogens can also contribute to CNS neuroprotection by promoting the anti-inflammatory M2 phenotype of microglia, which favors an increase in the percentage of CD19^+^CD9^+^IL-10^+^ regulatory B cells (175).

It is important to emphasize the importance of the protective effect of estrogens through the mechanisms of regulatory cells. In ovariectomized C57BL/6-EAE females, estrogen treatment improved the disease score and the percentage of regulatory B and T cells. Estrogens increase the percentage of CD8^+^CD122^+^ cells, CD19^+^CD5^+^ CD1d^high^ cells, CD19^+^ TIM-1^+^ cells and CD19^+^ CD138^+^ CD44^high^ B cells in both males and females but increase the percentage of CD19^+^ PD-L1^high^ B cells in females only (180). These studies clearly revealed that B cells are essential for providing estrogen-regulated protection against MS, indicating that estrogens act directly on B cells to positively modulate their function.

In contrast to estrogen, PRL is considered a hormone that promotes the development of this disease. MS patients in remission and with active disease presented higher serum PRL concentrations and greater *ex vivo* and *in vitro* Jak/Stat pathway signaling in B cells than healthy individuals. PRL also increased the expression of BAFF and Bcl2 *in vitro* (5-50 ng/mL). Therefore, B cells from patients with MS and hyperprolactinemia are less susceptible to apoptosis. Similarly, PRL increases the production of cells that produce anti-MOG antibodies that induce demyelination and correlates with the serum level of PRL in patients with MS (181). Like in SLE, it is suggested that PRL favors the maturation of autoreactive clones of B cells that evade central tolerance mechanisms and induce the maturation of autoantibody-producing cells.

In mouse models, PRL-producing B cells infiltrating chronic/late EAE lesions in C57BL/6 mice play a proinflammatory role in the CNS. B cells express PRL and GH mRNA, but only PRL markedly increases the expression of the T-box transcription factor eomesodermin (Eomes) in CD4^+^ T cells, which exacerbates the manifestations of EAE by promoting cytotoxic effects (182, 183). CD4^+^Eomes^+^ cells promote granzyme B production and lysosomal degranulation (183). Furthermore, in the coculture of T-B cells, an increase in CD4^+^CD107a^+^ Eomes^+^ T cells was observed, where CD107a^+^ was also an index of continuous and cytotoxic lysosomal degranulation. Similarly, PRL production in B cells is related to the expression of the transcription factor Zbtb20, which originally positively regulates pituitary PRL production; its inhibition reduces the development of EAE and the infiltration of CD4^+^CD107a^+^Eomes^+^ T cells (182).

Strong evidence for the antagonistic effects of both estrogens and PRL in MS has been shown. Estrogens confer protection through the induction of regulatory B cells or those with high expression of PDL-1, which reduces the proliferation of effector T cells; PRL appears to increase the incidence of MS, promoting the survival of B cells and increasing the cytotoxic activity of T cells (**Figures 4A, B**). This antagonism would explain the controversial role of hormones in the course of MS in pregnancy and in fertility treatments, which are both characterized by increases in PRL and estrogen levels.

## Conclusion

Hormones interact with their receptors are involved in the development, activation, and differentiation of B cells. It is important to mention that their effects also depend on the stage of cell maturation and not only on the concentration. Different hormones, such as PRL, GH, and P4, are secreted by B cells and express the receptor for PRL, estrogen, GH, testosterone, and P4 (**Figures 2**, **3**; **Table 1**). Hormone signaling, together with other signals from the immune response in B cells, can regulate multiple mechanisms in these cells. However, their role in the bidirectional communication network between the endocrine system and immune system is complex but should not be addressed as an isolated system in nature. Considering the development of autoimmune diseases that we address here and given that B cells play a major role in the onset of these diseases, the hormonal effect on B-cell function could explain the sexual dimorphism characteristic of autoimmune diseases. Some hormonal abnormalities may be related to the exacerbation of autoimmune disease, as is the case for estrogen, PRL and probably GH, which are fundamental for the progression and severity of these pathological processes, while testosterone and P4 may have protective effects. This finding provides insight into the search for more effective diagnostic and therapeutic options.

In this regard, the current challenge must be focused on proposing options that enable the identification of specific hormonal abnormalities and the cell populations involved in patients who are at risk. In addition, further studies of this immunoendocrine duality are needed.

## Author contributions

PS-S: Software, Supervision, Methodology, Formal analysis, Data curation, Writing – review & editing, Writing – original draft, Investigation, Conceptualization. RV-G: Methodology, Investigation, Writing – review & editing, Writing – original draft, Supervision. ML-H: Methodology, Conceptualization, Writing – review & editing, Writing – original draft, Supervision, Investigation. LC-S: Writing – review & editing, Writing – original draft, Supervision, Investigation. AC-R: Writing – review & editing, Writing – original draft, Visualization, Validation, Supervision, Software, Resources, Project administration, Methodology, Investigation, Funding acquisition, Formal analysis, Data curation, Conceptualization.
